# Multivariate physiological recordings in an experimental hemorrhage model

**DOI:** 10.1016/j.dib.2018.01.053

**Published:** 2018-01-31

**Authors:** Farid Yaghouby, Chathuri Daluwatte, Nicole R. Marques, Muzna Khan, Michael Salter, Jordan Wolf, Christina Nelson, John Salsbury, Perenlei Enkhbaatar, Michael Kinsky, David G. Strauss, George C. Kramer, Christopher G. Scully

**Affiliations:** a Office of Science and Engineering Laboratories, Center for Devices and Radiological Health, US Food and Drug Administration, Silver Spring, MD, United States; b Department of Anesthesiology, The University of Texas Medical Branch, Galveston, TX, United States; c Office of Clinical Pharmacology, Office of Translational Sciences, Center for Drug Evaluation and Research, US Food and Drug Administration, Silver Spring, MD, United States

## Abstract

In this paper we describe a data set of multivariate physiological measurements recorded from conscious sheep (*N* = 8; 37.4 ± 1.1 kg) during hemorrhage. Hemorrhage was experimentally induced in each animal by withdrawing blood from a femoral artery at two different rates (fast: 1.25 mL/kg/min; and slow: 0.25 mL/kg/min). Data, including physiological waveforms and continuous/intermittent measurements, were transformed to digital file formats (European Data Format [EDF] for waveforms and Comma-Separated Values [CSV] for continuous and intermittent measurements) as a comprehensive data set and stored and publicly shared here (Appendix A). The data set comprises experimental information (e.g., hemorrhage rate, animal weight, event times), physiological waveforms (arterial and central venous blood pressure, electrocardiogram), time-series records of non-invasive physiological measurements (SpO_2_, tissue oximetry), intermittent arterial and venous blood gas analyses (e.g., hemoglobin, lactate, SaO_2_, SvO_2_) and intermittent thermodilution cardiac output measurements. A detailed explanation of the hemodynamic and pulmonary changes during hemorrhage is available in a previous publication (Scully et al., 2016) [1].

**Specifications table**

**Table:** 

Subject area	*Physiology*
More specific subject area	*Multivariate physiological monitoring: hemodynamic, cardiovascular and pulmonary variables*
	*Critical care monitoring*
Type of data	*Digitized times series in European Data Format (EDF) and CSV*
How data was acquired	*Continuous waveforms and variables were recorded using a data acquisition system through invasive transducers or noninvasive electrode sensors. Intermittent measurements from laboratory blood gas analyses were also recorded by technicians and transformed to digital formats. All data were synchronized and consolidated into a zip file with a specific time stamp for each time series entry.*
Data format	*Raw synchronized and combined into EDF and CSV formats*
Experimental factors	*Data were continuously recorded in consecutive phases throughout the course of the experiment: baseline, hemorrhage, post-hemorrhage, transfusion and post-transfusion. Interventions were applied during hemorrhage and transfusion phases in which blood has been drawn or re-injected back to the animal, respectively. Each animal underwent two hemorrhages separated by at least 3 days at two different hemorrhage rates (1.25 ml/kg*_*BW*_*/min or 0.25 ml/kg*_*BW*_*/min).*
Experimental features	*Data recorded from large animals during experimental hemorrhages at two different rates. A wide range of continuous and intermittent measurements has been acquired from each animal to reflect physiological changes and variabilities with response to hemorrhage.*
Data source location	*Data was originally recorded at Department of Anesthesiology, University of Texas Medical Branch, Galveston, Texas, USA.*
Data accessibility	*Data is shared to be publicly available for users in this article (*Appendix A).

**Value of the data**

• To investigate the effects of hemorrhage rate on various physiological system responses in an animal model.
• To evaluate the performance of physiological measurements estimated from waveform analysis algorithms in continuous monitoring of patient status during acute hemorrhage.
• To develop novel biomarkers and smart monitoring indices of hemorrhage using continuous measurements and machine learning algorithms compared to standard clinical measurements such as blood gas analysis.
• To develop analytical algorithms for physiological waveform feature detection or signal quality assessment under stable and unstable physiological conditions.

## 1 Data

This data set includes physiological waveforms, continuous variables and intermittent laboratory measurements of blood samples and cardiac output estimations acquired during experimental hemorrhage in an ovine model. Physiological waveforms and continuous variables were recorded using different devices at variable sampling rates (Table 1). However, all recordings were consolidated, synchronized and stored to the final digital format in which a unique time vector was assigned to each variable.

**Table 1:** Description of physiological waveform signals and continuous variables recorded in each animal.

Type	Recording	Sensor and locations	Data Acquisition System	Sampling time
Physiological waveform signals	Arterial blood pressure (ABP)	Polyvinylchloride catheter implanted at right femoral artery	PowerLab (ADInstruments)	250 samples per second^a^
	Pulmonary arterial pressure (PAP)	Swan-Ganz catheter implanted at common pulmonary artery	PowerLab (ADInstruments)	250 samples per second^a^
	Central venous pressure (CVP)	Swan-Ganz catheter implanted at common pulmonary artery	PowerLab (ADInstruments)	250 samples per second^a^
	Electrocardiogram (EKG)	Ag-AgCl electrode patches secured to lower limbs and left rear limb	PowerLab (ADInstruments)	250 samples per second^a^
				
Continuous variables	Peripheral oxygen saturation (SpO_2_)	Pulse-oximeter probe secured to tail	Masimo Radical-7	One sample per every 2 s
	Cerebral tissue oximetry (rSO_2-cerebral_)	NIRS optical tissue oximetry secured to forehead	Nonin SenSmart Model X-100	One sample per every 4 s
	Thigh muscle tissue oximetry (rSO_2-thigh_)^b^	NIRS optical tissue oximetry secured to thigh muscle	Nonin SenSmart Model X-100	One sample per every 4 s
	Urinary output rate (UO rate)	Foley catheter implanted at urinary bladder	Bard Medical	One sample per every 60 s

a The original sampling rate of 1000 Hz has been reduced to 250 Hz.

b rSO_2-thigh_ was measured from two thigh locations on the same side in all animals.

## 2 Experimental design, materials and methods

Data was acquired from adult female sheep (*N* = 8; 37.4 ± 1.1 kg) under a protocol approved by the Institutional Animal Care and Use Committee at the University of Texas Medical Branch. After a 15-day quarantine period for medical examinations and adaptation to the environment, each animal was surgically prepared in a sterile operating room. To implant recording catheters and transducers, anesthesia was initiated by injecting 5 mg/kg ketamine (KetaVed; Vedco Inc., St. Joseph, MO) and maintained during the surgery with a mixture of 2–5% isoflurane (Piramal Healthcare Andhra Pradesh, India) in oxygen.

Arterial and venous lines were implanted in left and right femoral vessels to continuously record arterial blood pressure (ABP) and blood sampling; a 7F Swan-Ganz thermodilution catheter (131F7; Edwards Life Science, Irvine, CA) was placed into the common pulmonary artery to record pulmonary arterial pressure (PAP) and central venous pressure (CVP) as well as intermittent cardiac output. To avoid blood clotting, implanted lines and catheters were connected to a transducer (Truwave PX4 × 4; Edwards Life Science, Irvine, CA) and continuously flushed with heparinized saline (~3 mL/h per line). Following surgery completion, sheep were monitored for core body temperature, complete blood cell count, and any signs for discomfort, and pain. Buprenorphine (Buprenorphine SR; ZooPharm, Laramie, WY) was administrated before and after surgery for analgesia. During recovery, maintenance lactated Ringer's solution was used (2 mL/kg_BW_/h) for resuscitation. There was a 7 day recovery period following surgery.

**Fig. 1:**
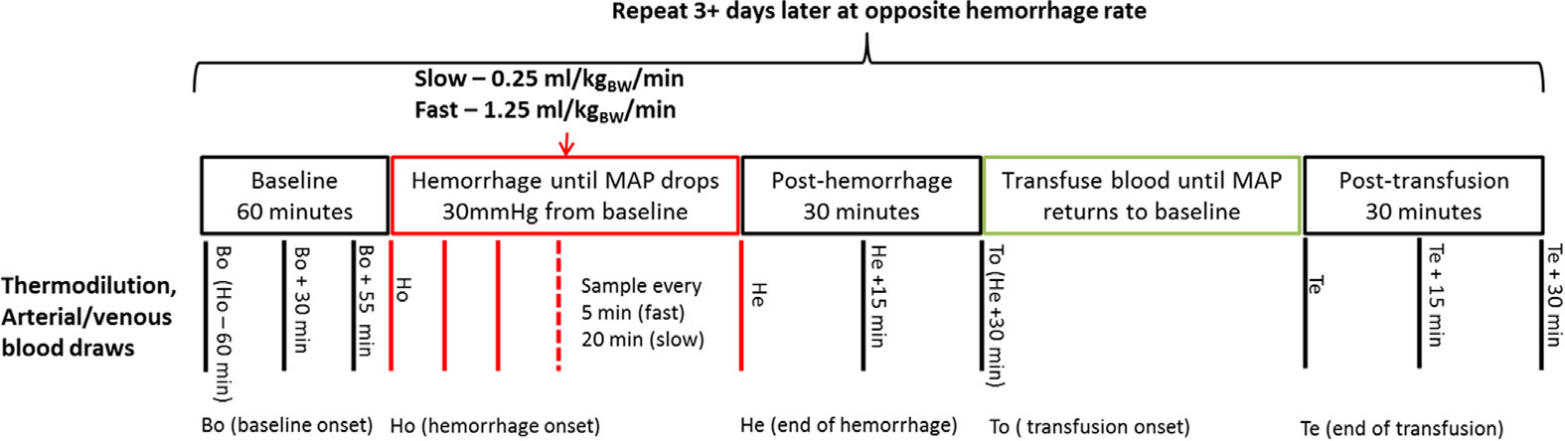
Timeline for the experimental protocol. Each animal is monitored through baseline, hemorrhage and recovery periods with a full range of continuous and intermittent physiological measurements. Bo: baseline onset; Ho: hemorrhage onset; He: end of hemorrhage; To: transfusion onset; and Te: end of transfusion.

Following the recovery period, experimental hemorrhages were randomly induced to each sheep at fast (1.25 mL/kg_BW_/min) or slow (0.25 mL/kg_BW_/min) rates using a large gauge sterile tubing rotary pump (MasterFlex Model 7518-10; Cole-Parmer, Vernon Hills, IL). The experiment was started following sensor placement (non-invasive electrode patches were secured for EKG, pulse oximeter and tissue oxygenation) and instrumentation setup. Data was recorded during baseline (60 min), hemorrhage (experiment dependent), post-hemorrhage (30 min), blood transfusion (experiment dependent) and post-transfusion (30 min) periods, Fig. 1. MAP was continuously monitored during the hemorrhage and the hemorrhage was terminated when a 30 mmHg drop in MAP was observed compared to baseline MAP. During transfusion, blood was reintroduced until the animal's MAP was restored to baseline. Following the post-transfusion period (after data recording ended) on the first day of experiment the remaining blood was reinfused. Three (or more) days later, the experiment was repeated on each animal at the alternate hemorrhage rate of the first day [1].

**Fig. 2:**
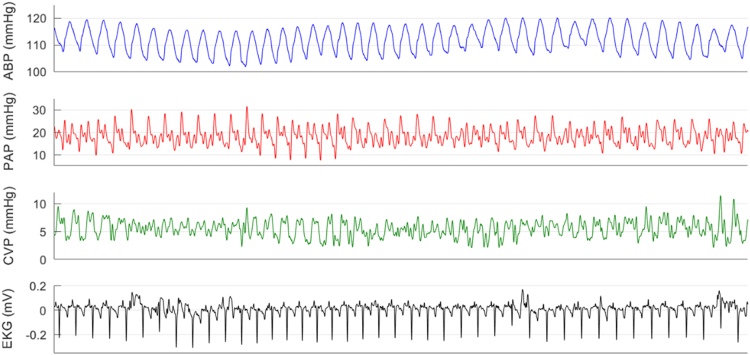
A 20 s sample from an EDF recording: ABP (blue), PAP (red), CVP (green) and EKG (black) waveforms are shown for animal 40_1.

Continuous physiological waveforms including ABP, PAP, CVP and EKG were recorded (sampling rate: 1000 Hz) using Powerlab data acquisition system and LabChart 7 Pro (ADInstruments Inc., Colorado Springs, CO) on a PC. Waveforms were converted to EDF format using LabChart 7 Pro and then downsampled to 250 Hz using a 4th order anti-aliasing filter, Fig. 2 (EDFbrowser 1.6; http://www.teuniz.net/edfbrowser). Continuous variables including SpO_2_, regional tissue oximetry and urinary output were recorded using Masimo Radical-7 pulse oximeter (Masimo, Irvine, CA), Nonin SenSmart Model X-100 (Nonin Medical, Inc., Plymouth, MN) and Foley catheter (Bard Medical, Covington, GA), respectively at different sampling rates (Table 1). At the end of experiment, recorded variables were downloaded from each device to a computer and converted to CSV.

Intermittent measurements including arterial and venous blood draws and thermodilution cardiac output measurements were made at fixed time points during the baseline, post-hemorrhage, and post-transfusion periods (noted by the vertical lines in Fig. 1). During the fast hemorrhage, measurements were made every 5 min and during slow hemorrhage measurements were made every 20 min. (Table 2 and Fig. 1). To estimate cardiac output, a 10-mL iced saline bolus was first injected into the right artery; two or three thermodilution measurements of cardiac output were recorded using the pulmonary artery catheter. The average value for recorded cardiac outputs was saved. Arterial and venous blood samples were taken from femoral artery and mixed venous blood, respectively, in 1 cc heparinized tuberculin syringe and analyzed using a Siemens RAPIDPoint 500 (Siemens, Malvem, PA) for measurement of blood gas, electrolytes, metabolites and CO-oximetry variables including but not limited to hematocrit, total hemoglobin, oxygen saturation (SO_2_), partial pressure of oxygen (PO_2_), partial pressure of carbon dioxide (PCO_2_), bicarbonate (HCO_3_), base excess, pH, and lactate. 12 h prior to the experiment, water and maintenance fluids were removed but food was provided. During the experiment animals were able to sit and stand in the cage and were closely monitored for any symptoms or signs of discomfort. Animals were euthanized using deep anesthesia following the second experimental day [1].

**Table 2:** Description of intermittent measurements.

Type	Sample	Measurements	Device	Sampling time
Blood-gas analysis	Arterial samples	Blood gas, Electrolytes, Metabolites and CO-oximetry	Blood gas system (Siemens RAPIDPoint 500)	Variable^a^
	Mixed venous samples	Blood gas, Electrolytes, Metabolites and CO-oximetry	Blood gas system (Siemens RAPIDPoint 500)	Variable^a^
Cardiac output	Thermodilution measurement	Two or three measurements were made at each time point and averaged for a single cardiac output value	Swan-Ganz thermodilution catheter	Variable^a^

a 60 min, 30 min, and 5 min before the start of hemorrhage, every 5 min (fast hemorrhage) or 20 min (slow hemorrhage) during the hemorrhage, 15 min and 30 min after the end of hemorrhage, and 15 min and 30 min after re-transfusion of blood.

Simultaneously recorded data during different phases of the experiment were then digitized, re-arranged and stored as binary EDF or CSV file. Details for each specific recording are shown in Table 3.

**Table 3:** Format and content for stored files.

Type	File name	Format	variables	comments
Experiment information	Info	CSV	Animal ID	General information about the experiment including but not limited to timing, animals, experimental treatments, etc.
			Animal weight	
			Hemorrhage rate	
			Event times	
				
Waveforms	Animal ID^a^_waveforms	EDF	Header	Header file includes animal ID, sampling rate, start date and time, and information about each waveform (labels, units, physical ranges, etc.)
			ABP, PAP, CVP, and EKG time series	
				
Continuous variables	Animal ID_ SpO2	CSV	Time	Continuous (low-resolution) SpO_2_ sampled at 0.5 Hz by Masimo pulse oximeter.
			SpO_2_	
	Animal ID_ rSO2	CSV	Time	Continuous (low-resolution) regional oxygenations sampled at 0.25 Hz by Sensmart.
			rSO_2__cerebral, rSO_2__thigh1, rSO_2__thigh2	
	Animal ID_ UO	CSV	Time	Continuous estimation of urinary output rate.
			Urinary Output rate	
				
Intermittent measurements	Animal ID_Arterial blood	CSV	Events, Time	Blood-gas analysis results and timings from arterial samples.
			pH, PCO_2_, pO_2_, HCO_3_, BE, Hct, tHb, sO_2_, FO_2_Hb, FCOHb, FmetHb, FHHB, Na, K, Ca, Cl, Glu, Lac^b^	
	Animal ID_ Venous blood	CSV	Events, Time	Blood-gas analysis results and timings from venous samples.
			pH, PCO_2_, pO_2_, HCO_3_, BE, Hct, tHb, sO_2_, FO_2_Hb, FCOHb, FmetHb, FHHB, Na, K, Ca, Cl, Glu, Lac	
	Animal ID _CO	CSV	Event, Time	Estimated CO (average of repeated 3 measurements)
			Thermodilution estimated cardiac output	

a Animal ID format: “Sheep#_$” in which # refers to the assigned number to each animal (e.g. 40, 229, etc.) and $ refers to the experiment day (1 or 2).

b pH, potential of hydrogen; PCO_2_, Partial Pressure of Carbon Dioxide; pO_2_, Partial Pressure of Oxygen; HCO_3_, Bicarbonate; BE, base excess; Hct, hematocrit; tHb, total hemoglobin; sO_2_, Oxygen saturation; FO_2_Hb, fraction of oxyhemoglobin; FCOHb, fraction of carboxyhemoglobin; FmetHb, fraction of methemoglobin; FHHB, fraction of deoxyhemoglobin; Na, sodium; K, potassium; Ca, calcium; Cl, chloride; Glu, glucose; Lac, lactate.

Not all signals were successfully recorded during all experiments. Particularly, regional oximetry measurements from thigh locations frequently dropped out during the experiments. File MissingData.csv in the zip folder includes a table noting which data is available for each experiment.

## Footnotes

Appendix A Supplementary data associated with this article can be found in the online version at 10.1016/j.dib.2018.01.053.
